# *In Vitro* Evaluation of the Antioxidant Activity and Wound Healing Properties of Jaboticaba (*Plinia peruviana*) Fruit Peel Hydroalcoholic Extract

**DOI:** 10.1155/2016/3403586

**Published:** 2016-08-18

**Authors:** Heloisa da S. Pitz, Aline Pereira, Mayara B. Blasius, Ana Paula L. Voytena, Regina C. L. Affonso, Simone Fanan, Adriana C. D. Trevisan, Rosa M. Ribeiro-do-Valle, Marcelo Maraschin

**Affiliations:** Plant Morphogenesis and Biochemistry Laboratory, Federal University of Santa Catarina, 1346 Admar Gonzaga Road, 88048-000 Florianópolis, SC, Brazil

## Abstract

Jaboticaba is a fruit from a native tree to Brazil,* Plinia peruviana*. Jaboticaba peels are an important source of antioxidant molecules such as phenolic compounds. This study aimed to evaluate* in vitro* the activity of a hydroalcoholic extract of jaboticaba fruit peels (HEJFP) in wound healing processes and antioxidant activity in murine fibroblasts (L929 cell line). HEJFP concentrations (0.5, 1, 5, 10, 25, 50, 100, and 200 *µ*g/mL) were tested in MTT assay and cell proliferation was verified at 100 *µ*g/mL after 24 h and at 25, 50, and 100 *µ*g/mL after 48 h of extract exposure. Evaluation of antioxidant activity was performed at 0.5, 5, 25, 50, and 100 *µ*g/mL HEJFP concentrations. Cell treatment with HEJFP at 25, 50, and 100 *µ*g/mL for 24 h followed by H_2_O_2_ exposure for 3 h showed a strong cytoprotective effect.* In vitro* scratch wound healing assay indicated that none of tested HEJFP concentrations (0.5, 5, 25, 50, and 100 *µ*g/mL) were capable of increasing migration rate after 12 h of incubation. These results demonstrate a positive effect of HEJFP on the wound healing process on L929 fibroblasts cell line, probably due to the antioxidant activity exhibited by phytochemicals in the extract.

## 1. Introduction

Wound healing is a process divided into three interactive and overlapping phases classified as inflammation, tissue formation, and tissue remodeling [1]. During inflammation, neutrophils and monocytes invade the injury tissue and start to secrete proteolytic enzymes, proinflammatory cytokines, and growth factors. Besides, these cells also secrete reactive oxygen species (ROS), important molecules that defend the body against bacteria and microorganism invasion [2].

The next phase known as tissue formation is characterized by proliferation and migration of fibroblasts and keratinocytes from the wound edge to the wound bed [3]. Angiogenesis is triggered and leads to the formation of granulation tissue, which is important to support the nutrients and oxygen supply in injured tissue [4–6]. In this tissue, fibroblasts become myofibroblasts which synthesize and deposit extracellular matrix (ECM) compounds, especially collagen. Besides, these cells are responsible for wound contraction and maturation of the granulation tissue [7].

At remodeling phase, there is a reduction on cellularity due to the apoptosis of myofibroblasts, endothelial cells, and inflammatory cells. The synthesis of ECM is reduced and ECM's components are modified as the matrix is remodeled [8].

Impaired wound healing is a problem that may be caused by uncontrolled inflammatory and immune responses, microbial infection, and excessive ROS production [9]. Excessive amounts of ROS may modify and/or degrade ECM proteins and damage dermal fibroblasts and keratinocytes functions. Besides, ROS-mediated transcription causes the maintained proinflammatory cytokines secretion and induction of matrix metalloproteases [10].

Jaboticaba is a fruit from a native tree to Brazil, that is,* Plinia peruviana*. Studies have shown important biological properties of anthocyanins, mainly those related to anti-inflammatory activity and antioxidative stress [11]. Jaboticaba fruit peels are the main source of anthocyanins in the fruit and it has been used in traditional medicine to treat diarrhea, skin irritation, hemoptysis, and asthma [12–14]. This study aimed to evaluate the antioxidant activity of a HEJFP and its role in wound healing processes as migration and proliferation of murine fibroblasts (L929 cell line).

## 2. Materials and Methods

### 2.1. Plant Material Collection and Extraction

Fruits of* P. peruviana* were collected from a backyard format planting system during the harvest season (spring, 2014) in Guaxupé, Minas Gerais, Brazil. The plant was authenticated by Dr. Marcos Sobral and a voucher specimen (FLOR 55902) was preserved at FLOR herbarium (Department of Botany, Federal University of Santa Catarina, Florianópolis, southern Brazil).

The fruit peels of jaboticaba were lyophilized and powdered by an electric grinder. The dried and powdered biomass was added to 50% ethanol solution (v/v), pH 3.6 (1 : 10 w/v). The mixture was microwaved (three pulses of five seconds with 60 seconds of interval between each of the pulses) to extract the compounds of interest. The HEJFP was recovered by filtration on cellulose membranes under vacuum.

### 2.2. Determination of Total Phenolic Content

The total phenolic content of HEJFP was measured spectrophotometrically [15]. For that, HEJFP was diluted in 50% ethanol solution, pH 3.6 (1 : 10 v/v). Subsequently, 1 mL of HEJFP previously diluted was added to 5 mL 95% methanol solution. After this second dilution, sample (1 mL) was added to 1 mL 95% ethanol solution, 5 mL distilled water, and 0.5 mL Folin-Ciocalteu's reagent and incubated for 7 min.

After incubation, 1 mL 5% sodium carbonate solution was added and kept in the darkness at room temperature for 1 h. A blank solution was prepared as described above replacing the sample by 50% of ethanol solution, pH 3.6. The absorbance was measured at 725 nm, using a UV-Vis spectrophotometer (BEL LGS 53, BEL Engineering, Monza, Italy).

The total phenolic compounds were quantified using a standard curve of gallic acid. The results were expressed as mg gallic acid equivalents/g dry weight of jaboticaba biomass.

### 2.3. Determination of the Total Flavonoid Content

The determination of total flavonoids was based on aluminum chloride colorimetric method [16].

Previously, HEJFP was diluted in 50% ethanol solution, pH 3.6 (1 : 10 v/v). 0.5 mL of diluted HEJFP was added to 2.5 mL ethanol and 0.5 mL 2% aluminum chloride diluted in methanol and incubated for 1 h. A blank solution was prepared as described above replacing the sample by 50% of ethanol solution, pH 3.6. The absorbance was measured at 420 nm in a UV-Vis spectrophotometer (BEL LGS 53, BEL Engineering, Monza, Italy). The quantification of total flavonoids was carried out using a quercetin standard curve. The results were expressed as mg quercetin equivalents/g dry weight of jaboticaba biomass.

### 2.4. Antioxidant Activity (DPPH Assay)

The 2,2-diphenyl-2-picrylhydrazyl (DPPH) assay is a chemical method that measures the capacity of a compound to scavenge free radicals based on the decrease in absorbance during the reaction [17]. A stock solution of 0,0079 g of DPPH was diluted in 2.5 mL methanol. This solution was further diluted in a concentration of 1 : 100 (v/v) in 80% methanol (v/v). The absorbance of this DPPH solution should be around 0.5 and 0.6. The HEJFP, previously diluted in 50% ethanol, pH 3.6, at 1 : 100 (v/v), was added to DPPH/80% methanol solution (1 : 30 v/v). The capacity of the HEJFP to inhibit DPPH radicals was measured spectrophotometrically at 515 nm, after incubation for 5, 10, 20, 30, 40, and 50 min in the dark, at room temperature. The same procedure described above was used to test the 50% ethanol, pH 3.6, solution to ensure that the solvent was not reacting with DPPH/80% methanol solution. The percentage of inhibition of DPPH radicals was calculated by the following formula (Abs. = absorbance):

(1) inhibition  DPPH  %=Abs.  DPPH/80%  methanol  solution−Abs.  HEJFPAbs.  DPPH/80%  methanol  solution×100.

### 2.5. Cell Proliferation and Viability Assay Using L929 Fibroblast

L929 mouse fibroblast cells were seeded at a density of 5 × 10^3^ cells/well into a 96-well plate in DMEM culture medium supplemented with 10% FBS and incubated at 37°C, in a humidified 5% CO_2_ atmosphere overnight. After incubation, DMEM was replaced by DMEM 10% FBS containing 0.5, 1, 5, 10, 25, 50, 100, and 200 *μ*g/mL (dry weight) of HEJFP, except in control, where the culture medium was replaced by fresh DMEM. Cells were incubated for 24 h and 48 h, at 37°C, in a humidified 5% CO_2_ atmosphere. Afterwards, the culture medium was replaced by 100 *μ*L of fresh DMEM along with 10 *μ*L 3-(4,5-dimethylthiazol-2-yl)-2,5-diphenyltetrazolium bromide (MTT) solution (5 mg/mL in PBS) per well and incubated in the dark, for 3 h, at 37°C, in a humidified 5% CO_2_ atmosphere. A negative control without cells with 100 *μ*L of DMEM and 10 *μ*L of MTT solution was required. Subsequently, 85 *μ*L of culture medium was removed and 50 *μ*L of DMSO was added onto each well and incubated for more 10 min, at 37°C, in a humidified 5% CO_2_ atmosphere. After homogenizing formazan crystals, the absorbance at 540 nm was determined by an ELISA plate reader. The percentage of cell proliferation/viability was calculated and compared to control (100% of viability).

### 2.6. Hydrogen Peroxide-Induced Oxidative Stress in L929 Fibroblast Cells and Evaluation of Cell Survival

Hydrogen peroxide was used for induction of oxidative stress as described by Balekar et al. [18] and Ponnusamy et al. [19]. The L929 fibroblast cells were seeded at a density of 5 × 10^3^ cells/well into a 96-well plate in DMEM supplemented with 10% FBS and incubated at 37°C, in a humidified 5% CO_2_ atmosphere overnight. A curve with H_2_O_2_ concentrations (0.0625, 0.125, 0.25, 0.5, and 1.0 mM) was built to determine H_2_O_2_ dose which decreases cell viability by 80% after 24 h of exposure using MTT assay. The chosen concentration was 1.0 mM of H_2_O_2_. Subsequently, L929 fibroblast cells were seeded at a density of 5 × 10^3^ cells/well into a 96-well plate containing DMEM culture medium supplemented with 10% FBS and incubated overnight at 37°C, in a humidified 5% CO_2_ atmosphere. After incubation, DMEM with 10% FBS containing 0.5, 5, 25, 50, and 100 *μ*g/mL (dry weight) of HEJFP was used to treat cells in different times as follows: (1) cells were treated for 24 h followed by 1.0 mM of H_2_O_2_ exposure for 3 h, (2) cells were exposed concomitantly to HEJFP and 1.0 mM of H_2_O_2_ for 24 h, and (3) cells were exposed to 1.0 mM of H_2_O_2_ for 3 h followed by cells treatment with HEJFP for 24 h. Evaluation of cell survival was performed using MTT assay as described above.

### 2.7. Scratch Assay

The stimulatory effect of HEJFP on migration of L929 cells was determined as described by Balekar et al. [18]. The L929 fibroblast cells were seeded at a density of 5 × 10^5^ cells/well into a 24-well plate containing DMEM culture medium supplemented with 10% FBS and incubated overnight at 37°C, in a humidified 5% CO_2_ atmosphere. After incubation, DMEM was completely removed and the adherent cell layer was scratched with a sterile yellow pipette tip. Cellular debris was removed by washing off with phosphate buffer saline (PBS). The cells were treated with DMEM with 10% FBS containing 0.5, 5, 25, 50, and 100 *μ*g/mL (dry weight) of HEJFP. Controls received only fresh DMEM. To avoid proliferation of cells, mitomycin C (10 *μ*g/mL) was added in each well along with control and HEJFP-treated cells; this way only migration was evaluated. The cells were incubated (at 37°C in humidified 5% CO_2_ atmosphere for 12 h) and then the recording of images of the scratch area was carried out in two different points, using a built-in camera in the microscope (40x magnification) at 0 h (just after scratching cells) and at 12 h after incubation with HEJFP and control. Data were analyzed with ImageJ 1.42q imaging software (National Institutes for Health, US) in order to determine the width of the scratch and thus to calculate the rate of migration of cells by the following formula:

(2) $$\begin{array}{c} \text{migration rate }\left(\;\%\;\right)=\frac{\text{distance within scratch }\left(0\;h\right)-\text{distance within scratch }\left(12\;h\right)}{distance\text{ within scratch }\left(0\;h\right)}\times 100. \end{array}$$

### 2.8. Statistical Analysis

Data were collected and summarized, followed by statistical analysis using one-way ANOVA and Tukey's test. *P* values lower than 0.05 were considered to be statistically significant. The values were expressed as mean ± SD or median as indicated in figures' captions.

## 3. Results

### 3.1. Total Phenolic and Flavonoid Contents of the Hydroalcoholic Extract

The total phenol and flavonoid contents of HEJFP were 92,2 ± 9,75 mg gallic acid equivalent/g and 6,43 ± 0,49 mg quercetin equivalent/g, respectively.

### 3.2. Antioxidant Activity

DPPH radical scavenging of HEJFP was measured in different times to determine the peak of antioxidant capacity. After 5 min of incubation, HEJFP inhibited 83.6% of DPPH radicals, showing an excellent antioxidant activity in few minutes of reaction. At 30 min of incubation, 91% scavenging activity was achieved and it remained until 50 minutes of reaction (Table 1).

### 3.3. Cell Proliferation and Viability

The effect of HEJFP on both cell proliferation and viability was evaluated in L929 murine fibroblasts cell line in different concentrations after 24 h and 48 h, using MTT assay.

**Figure 1:**
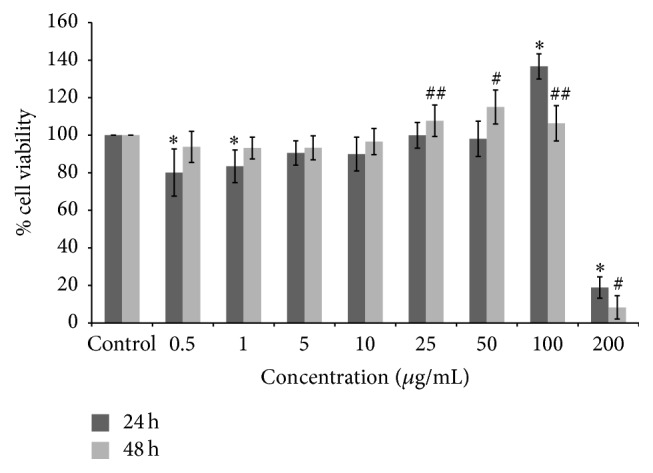
Percentage of survival of L929 fibroblast cells treated with HEJFP after 24 h and 48 h. Data are expressed as a mean ± SD (*n* = 18). *∗* indicates *P* < 0.01 against control for 24 h; # indicates *P* < 0.01 and ## indicates *P* < 0.05 against control for 48 h.

HEJFP was able to promote cell proliferation at 100 *μ*g/mL after 24 h and at 25, 50, and 100 *μ*g/mL after 48 h. The concentration of 200 *μ*g/mL was shown to be cytotoxic in both times of exposure, decreasing significantly cell viability. For the other concentrations assayed, the cell viability was higher than 80% (Figure 1).

### 3.4. Hydrogen Peroxide-Induced Oxidative Stress and Cell Survival

**Figure 2:**
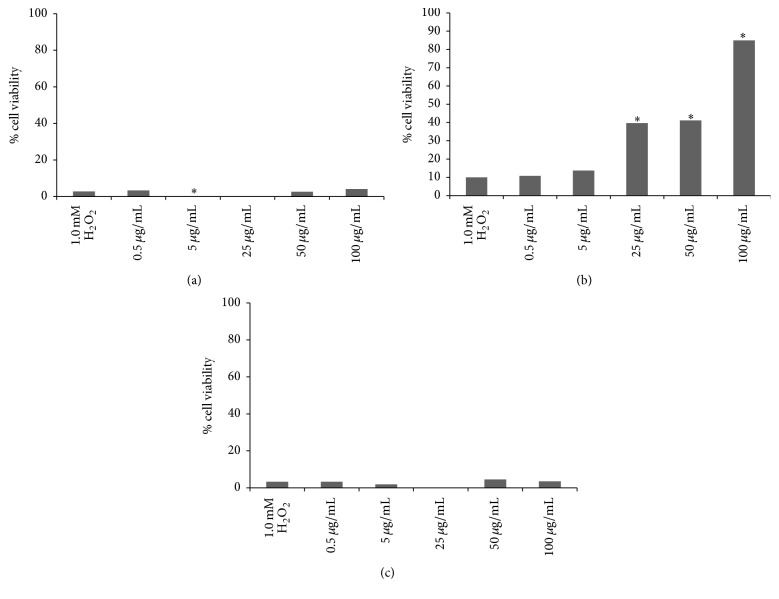
Viability (%) of cells treated with HEJFP after H_2_O_2_ exposure (a) and before H_2_O_2_ exposure (b) and concomitantly with H_2_O_2_ (c). Data are expressed as a median (*n* = 16). *∗* indicates *P* < 0.01 against 1.0 mM H_2_O_2_ control.

L929 fibroblast cells were treated with 1.0 mM H_2_O_2_ as a model study of oxidative stress and resulted in decrease of cell viability by 90% after 24 h of exposure. The antioxidant potential of the HEJFP was tested before and after H_2_O_2_ exposure for 3 h and concomitantly with H_2_O_2_ for 24 h. The HEJFP was not effective in protecting cells against oxidative stress before or concomitantly with exposure to H_2_O_2_, resulting in low rate of cell survival. However, when cells were first treated with HEJFP for 24 h, followed by H_2_O_2_ exposure for 3 h, the tested concentrations of 25, 50, and 100 *μ*g/mL protected the cells against adverse effects caused by H_2_O_2_-induced oxidative stress and maintained the cell viability (Figure 2).

### 3.5. Scratch Assay

**Figure 3:**
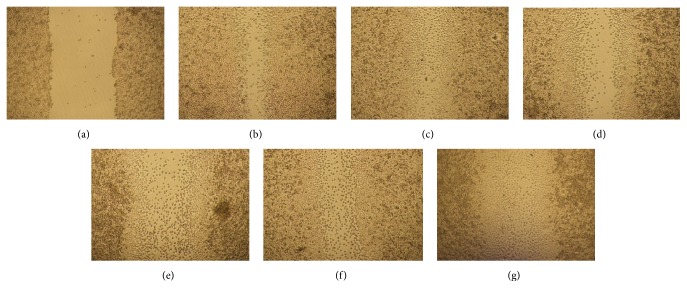
Microscopy images of L929 fibroblast cells migration after scratch ((a)—time 0) and after 12 h of HEJFP treatment. (b) Control, (c) 0.5 *μ*g/mL, (d) 5 *μ*g/mL, (e) 25 *μ*g/mL, (f) 50 *μ*g/mL, and (g) 100 *μ*g/mL.

L929 murine fibroblasts cell line was tested through the scratch assay to determine the capacity of these cells to migrate under HEJFP stimulus (Figure 3). L929 cells have a fast migration rate; then the time to evaluate the cell migration was determined as 12 h upon exposure to HEJFP. At 24 h, scratch is almost closed, making it difficult to analyze images.

Although concentrations of 0.5 and 100 *μ*g/mL increased the cell migration rate after 12 h, the effect was not significant when compared to control (Table 2).

## 4. Discussion

Plant extracts can be efficient in helping the wound healing process if they contain phytochemicals with antimicrobial and antioxidant activities and free radical scavengers and active compounds that enhance mitogenic activity, angiogenesis, collagen production, and DNA synthesis [20].

Jaboticaba fruit peel has a promising potential as a wound healing enhancer duo to its biomass rich in phenolic compounds. Indeed, those secondary metabolites have a well-known antioxidant activity that prevents tissue damage and stimulates wound healing [11, 21].

The evaluation of the effectiveness of HEJFP in wound healing process was performed* in vitro* using L929 murine fibroblasts cell line. Nowadays, cell culture is a popular and effective method to test the sensitivity of cells to selected groups present in the microenvironment. Fibroblasts cell cultures have been proposed as a method for testing wound healing activity* in vitro* [22].

Hydrogen peroxide-induced oxidative stress is an alternative to evaluate extract's antioxidant activity in cells. H_2_O_2_ is an important molecule in wound healing process, the effect of which shall be under control of a molecular antioxidant apparatus such as SOD, GPx, and phospholipid hydroperoxide glutathione peroxidase [23]. A cytoprotective effect of HEJFP was detected when oxidative stress was induced to cells after the treatment with the extract. In this sense, a plausible assumption takes into account the fact that the protective effect of the extract could be related to the antioxidant activity thereof, corroborated by the results obtained through DPPH assay.

Xu et al. [11] also found a protective effect against hydrogen peroxide-induced oxidative stress in keratinocytes and fibroblasts of black soybean seed coat extract. The authors assigned this effect to the antioxidant activity of anthocyanins, metabolites that belong to phenolic group and are found in abundance in jaboticaba fruit peels.

Cell proliferation and migration are two extremely important features during the tissue formation phase in the wound healing. Scratch assay is a form to mimic a wound* in vitro *and evaluate the cell migration rate. Once the cell monolayer is disrupted, the loss of cell-cell interaction results in increasing concentration of growth factors and cytokines at the wound edge, initiating migration and proliferation of cells [24]. Interestingly, although the HEJFP was not able to increase cell migration rate, the extracts at 25, 50, and 100 *μ*g/mL promoted fibroblasts proliferation. This mitogenic effect is a positive event for wound healing process because fibroblasts are important cells involved in wound contraction and ECM production [18].

## 5. Conclusion

 HEJFP has been shown to be* in vitro* a potential plant extract, enhancing the wound healing process. The cytoprotective effect of HEJFP in fibroblasts against hydrogen peroxide-induced oxidative stress can be assigned to its phenolic compounds, which have been proven to be strong antioxidants. Besides, HEJFP induced mitogenic activity of fibroblasts, an important feature in the wound healing process. Further investigations are necessary to isolate and identify the compounds responsible for these activities, as previous findings refer to the ellagic acid as a major compound in the HEJFP (unpublished data). Besides,* in vitro* studies measuring antioxidant enzymes will help understand the mechanisms underlying the effects described herein for the wound healing process.

## Figures and Tables

**Table 1:** Antioxidant activity of HEJFP determined by the DPPH assay.

Incubation time (min)	% inhibition of DPPH radical
5	83.6 ± 1.83
10	88.09 ± 1.52
20	90.18 ± 1.02
30	91.01 ± 0.42
40	91.36 ± 1.01
50	91.88 ± 1.28

Values are mean ± SD (*n* = 3).

**Table 2:** Scratch length (*µ*m) and cell migration rate (%) of L929 murine fibroblasts treated with HEJFP determined by the scratch assay.

	Length within the scratch (*µ*m)	% migration rate
Time 0	859.97 ± 113.9	0
Control	246.73 ± 62.84	71.31 ± 7.3
0.5 *µ*g/mL	157.56 ± 53.58	81.68 ± 6.23
5 *µ*g/mL	261.91 ± 99.32	69.54 ± 11.55
25 *µ*g/mL	263.36 ± 73.4	71.42 ± 8.53
50 *µ*g/mL	245.79 ± 138.65	69.37 ± 16.12
100 *µ*g/mL	183.31 ± 91.15	78.57 ± 10.6

Values are mean ± SD (*n* = 8).
